# *Mutant POLQ* and *POLZ/REV3L* DNA polymerases may contribute to the favorable survival of patients with tumors with POLE mutations outside the exonuclease domain

**DOI:** 10.1186/s12881-020-01089-9

**Published:** 2020-08-24

**Authors:** Fangjin Huang, Hisashi Tanaka, Beatrice S. Knudsen, Joanne K. Rutgers

**Affiliations:** 1 Department of Biomedical Sciences, Cedars-Sinai Medical Center, Los Angeles, CA 90048 USA; 2 Samuel Oschin Cancer Research Institute (SOCCI), Cedars-Sinai Medical Center, Los Angeles, CA 90048 USA; 3 Surgery, Cedars-Sinai Medical Center, Los Angeles, CA 90048 USA; 4 Pathology and Laboratory Medicine, Cedars-Sinai Medical Center, Los Angeles, CA 90048 USA; 5 Department of Pathology, University of Utah, Salt Lake City, UT 84112 USA

## Abstract

**Background:**

Mutations in the exonuclease domain of POLE, a DNA polymerase associated with DNA replication and repair, lead to cancers with ultra-high mutation rates. Most studies focus on intestinal and uterine cancers with POLE mutations. These cancers exhibit a significant immune cell infiltrate and favorable prognosis. We questioned whether loss of function of other DNA polymerases can cooperate to POLE to generate the ultramutator phenotype.

**Methods:**

We used cases and data from 15 cancer types in The Cancer Genome Atlas to investigate mutation frequencies of 14 different DNA polymerases. We tested whether tumor mutation burden, patient outcome (disease-free survival) and immune cell infiltration measured by ESTIMATE can be attributed to mutations in POLQ and POLZ/REV3L.

**Results:**

Thirty six percent of colorectal, stomach and endometrial cancers with POLE mutations carried additional mutations in POLQ (E/Q), POLZ/REV3L (E/Z) or both DNA polymerases (E/Z/Q). The mutation burden in these tumors was significantly greater compared to POLE-only (E) mutant tumors (*p* < 0.001). In addition, E/Q, E/Z, and E/Q/Z mutant tumors possessed an increased frequency of mutations in the POLE exonuclease domain (*p* = 0.013). Colorectal, stomach and endometrial E/Q, E/Z, and E/Q/Z mutant tumors within TCGA demonstrated 100% disease-free survival, even if the POLE mutations occurred outside the exonuclease domain (*p* = 0.003). However, immune scores in these tumors were related to microsatellite instability (MSI) and not POLE mutation status. This suggests that the host immune response may not be the sole mechanism for prolonged disease-free survival of ultramutated tumors in this cohort.

**Conclusion:**

Results in this study demonstrate that mutations in POLQ and REV3L in POLE mutant tumors should undergo further investigation to determine whether POLQ and REV3L mutations contribute to the ultramutator phenotype and favorable outcome of patients with POLE mutant tumors.

## Background

The analysis of thousands of cancers by The Cancer Genome Atlas (TCGA) consortium and academic institutions revealed a small group of cancers with mutations in *POLE* and an ultramutator phenotype [1, 2]. Multiple studies have found significantly improved survival in patients with *POLE* mutated endometrial cancers [1, 3–6], while the survival benefit was not as profound in *POLE* mutated colorectal carcinoma [7]. The *POLE* gene encodes the catalytic subunit of DNA polymerase epsilon, which catalyzes the leading strand synthesis during DNA replication. POLE possesses high-fidelity DNA polymerization, proofreading and 3′-5′ exonuclease activities, which promote accurate DNA synthesis [8]. First identified and reported in 2–6% of colorectal carcinomas [2, 9, 10], *POLE* mutations were also noted at frequencies of 6–9% amongst uterine corpus endometrial cancers [1, 11] and in gastric adenocarcinoma [12]. Mutations can be found across the entire *POLE* gene, but those in the *POLE* exonuclease domain are most prevalent in cancers with ultra-high mutation rates (> 100 mut/Mb). These cancers exhibit higher mutation rates than microsatellite instable (MSI) tumors associated with mismatch repair abnormalities. In addition, *POLE* ultra-mutant cancers also possess a high frequency of C-to-A transversions [13, 14]. A tumor associated inflammatory response, similar to that in MSI tumors, has been reported to occur early on during development of *POLE* mutated endometrial and colorectal cancers [15]. It is thought to be caused by neo-antigens that are generated as a result of the high mutation burden [16, 17] and render *POLE* mutant cancers responsive to immunotherapy [18].

Polymerase theta (POLQ) is a low-fidelity DNA polymerase lacking a 3′ to 5′ exonuclease function [19]. The enzyme is involved in the alternative non-homologous end-joining pathway (alt-NHEJ) [20], which is a backup mechanism of double stranded DNA break repair. This pathway predominates in cancer cells when other DNA repair pathways are missing or when telomere ends are deprotected [21, 22]. The loss of POLQ sensitizes cells to ionizing radiation and Polq-deficient mice exhibit increased DNA instability and genomic rearrangements, suggesting a role for POLQ as a guardian of the genome [23]. Both overexpression and loss of POLQ increase mutation frequencies [22, 24, 25]. Multiple structural motives in POLQ can interact with DNA, RAD51 and BRCA1 [26]. In addition, POLQ forms a complex with PARP-1 in a pathway of synthetic lethality with BRCA1 and is thus considered a therapeutic target [21, 22]. However, which domains in POLQ should be targeted remains to be determined [25].

REV3L (REV3 like, DNA directed polymerase zeta (POLZ) catalytic subunit) is involved in DNA synthesis that reads through damaged DNA (translesion DNA synthesis, TLS). The high efficiency of POLZ bypassing a broad spectrum of DNA lesions led to its recognition as a master TLS polymerase [27]. POLZ/REV3L has been linked to carcinogenesis in breast, lung, gliomas, and gastric cancers, and modulates cisplatin sensitivity [28–31].

Because studies describe overlapping functions and synergy among the over 14 DNA polymerases [27, 32, 33], we undertook an unbiased approach to identify mutations in polymerases within the TCGA tumor compendium. This analysis revealed a greater frequency of mutations in POLQ and POLZ/REV3L compared to other polymerases of similar gene length. Therefore, we propose that POLQ and POLZ/REV3L may cooperate with POLE in generating ultrahigh mutation rates.

## Methods

### Data acquisition

The data used in this study are based upon the whole exome sequence data sets generated by the TCGA Research Network: http://cancergenome.nih.gov/. The locations and frequencies of somatic mutations, MSI status and clinical stage and follow up information in TCGA Provisional datasets were obtained from cBioportal (http://www.cbioportal.org) [34, 35] up to 06/22/2016 (Supplementary Table 1). Mutation data were obtained by first selecting the “Query” tab from cBioPortal. The cancer type specific data were chosen from the “TCGA PanCancer Atlas Studies”. We entered the genes of interest, POLE, POLQ and REV3L to retrieve mutation data from the genes. When the result page was displayed, we accessed the information underneath the “Mutations” tab. We downloaded the number of mutations in each sample, the annotation of protein amino acid change, and the type of mutation. The survival information for each case (months after diagnosis) was obtained through the “Comparison/Survival” link. Functional domains of the proteins were provided by Pfam database [36]. Data visualization for mutations was performed with MutationMapper in cBioportal.

### Case selection criteria

We searched all cancer types in TCGA for those that possess 2 or more cases with *POLE* mutations. This yielded 15 tumor types that we named the PANCAN data set in this study. We then determined the frequency of mutations within additional polymerase within PANCAN. We selected the three adenocarcinomas (uterine corpus endometrial adenocarcinoma, colorectal adenocarcinoma, and stomach adenocarcinoma) with the highest frequency of double or triple mutated DNA polymerase status for more detailed analysis.

### Studies performed

Kaplan Meier survival plots were generated using clinical follow-up data available within the TCGA database. To determine the global mutational spectrum, we classified 6 types of nucleotide transitions or transversions. The frequency of each mutation type was calculated.

Digital images of all *E/Q* mutant tumors and representative cases of tumors with neither *POLE* nor *POLQ* mutations, and of MSI tumors were assessed by one author (JR) for the amount of tumor immune infiltrate. The combination of tumor infiltrating lymphocytes and the peritumoral lymphoid infiltrate was graded on a scale between 0 and 3. Tumor associated lymphocytes were graded as none (0), minimal (rare) to 1 per high powered field (HPF) (1), 2 to 5 per HPF (2) and > 5 per HPF (3). Peritumoral lymphocytes were assessed at the deepest advancing tumor front, graded at low power: none (0), minimal (1), mild (2), moderate (3) and marked (4). These visual semi-quantitative scores were compared with the immune scores evaluated using ESTIMATE (Estimation of STromal and Immune cells in MAlignant Tumor tissues using Expression data) [37]. ESTIMATE score were obtained from the website of ESTIMATE at MD Anderson: https://bioinformatics.mdanderson.org/estimate/disease.html. The platform type selected was RNA-Seq-V2. Data for colorectal, stomach and endometrial cancer types were downloaded. Next, we separated the colorectal, stomach and endometrial cancer cases into 3 quartiles defined by the highest 25%, intermediate 50%, and lowest 25% of mutational counts or MSI status, and calculated the immune scores for each group.

### Statistical data analysis

All statistical analyses were conducted in R v3.1.3. Plots were generated using ggplot2 package in R [38]. Data visualization methods were described previously [39]. The horizontal lines in the boxplots represent the 1st, 2nd and 3rd quartiles and whiskers outside the box show the 1.5 interquartile range. The significance of the differences of data illustrated in the boxplots was calculated using the Wilcoxon rank-sum tests. The Chi-square test was performed to test the significance of differences in frequencies of all tables. The significance in the Kaplan–Meier survival plot was calculated using the log rank test. Statistical significance was accepted at *p* < 0.05.

## Results

**Fig. 1:**
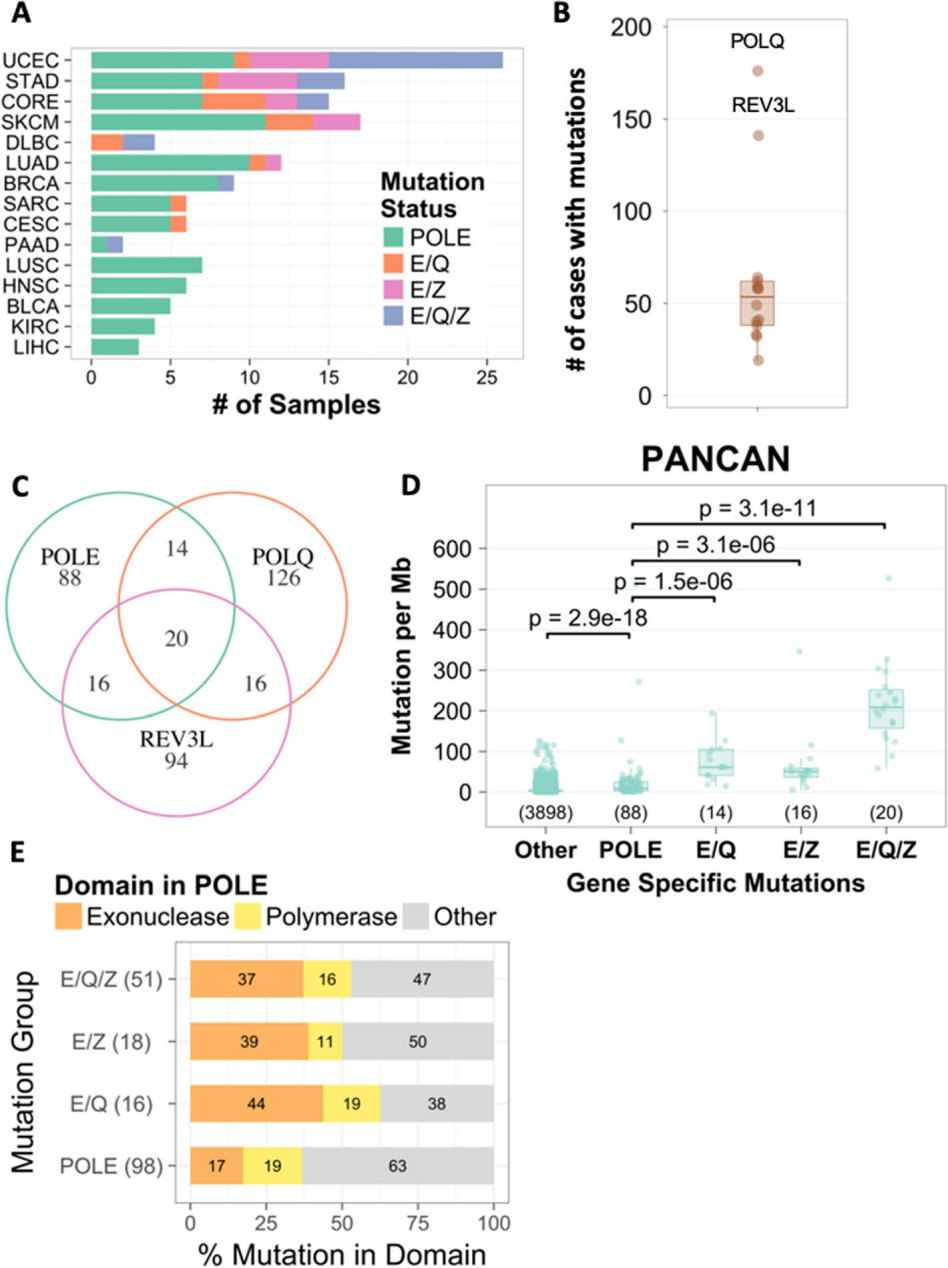
*Cancer types (PANCAN) with POLE/Q/Z mutations in TCGA.* **a** Number of cases with *POLE- only*, *E/Q*, *E/Z* and *E/Q/Z* mutations in 15 cancer types (cohort referred to as PANCAN) within TCGA. The x-axis shows the actual number of cases with *POLE* (green), *E/Q* (orange), *E/Z* (pink) and *E/Q/Z* (blue) mutations. The y-axis displays the 15 cancer types: Uterine corpus endometrial carcinoma (UCEC), Stomach adenocarcinoma (STAD), Colon and rectum adenocarcinoma [38], Skin cutaneous melanoma (SKCM), Lymphoid Neoplasm Diffuse Large B-cell Lymphoma (DLBC), Lung adenocarcinoma (LUAD), Breast invasive carcinoma (BRCA), Sarcoma (SARC), Cervical squamous cell carcinoma and endocervical adenocarcinoma (CESC), Pancreatic adenocarcinoma (PAAD), Lung squamous cell carcinoma (LUSC), Head and neck squamous cell carcinoma (HNSC), Bladder urothelial carcinoma (BLCA), Kidney renal clear cell carcinoma (KIRC), and Liver hepatocellular carcinoma (LIHC). **b** Case numbers with mutations in polymerase genes. The number of cases in PANCAN with mutations in the following polymerases is displayed on the Y-axis: DNTT, POLA1, POLB, POLD1, POLG, POLH, POLI, POLK, POLL, POLM, POLN, POLQ, REV1, REV3L. **c** Venn diagram displaying the number of cases in PANCAN with mutations in 1, 2 or 3 POL genes. **d** Mutations per Mb (y-axis) of PANCAN cases without *POLE* mutations (other) or with *POLE*, *E/Q*, *E/Z* and *E/Q/Z* (x-axis) mutations. Number of cases in each group are listed in parenthesis. **e** Mutation frequencies in *POLE* exonuclease and polymerase domains as a percentage of total number of mutations in the POLE exome. “Other” refers mutations in the entire Exome outside the exonuclease or polymerase domains. The cases are grouped by their polymerase mutation status on the y-axis, and the number in parenthesis represents the total number of POLE mutations within each group

Of the 33 cancer types in the TCGA database [40], we identified 15 cancer types (PANCAN) with 2 or more cases that possessed mutations in the *POLE* protein coding region (Fig. 1A and Supplementary Table 1). These 15 cancer types contained 138 cases with *POLE* mutations anywhere in the exome. 53% of these POLE mutant tumors carried mutations in one or more of the 14 other DNA polymerases. We observed DNA polymerases *POLQ* and *POLZ/REV3L* to be most frequently mutated (Fig. 1b). These two polymerases were mutated in 36% of tumors with *POLE* mutations. In fact, these two polymerases were even more commonly mutated than POLE in our PANCAN cohort (Supplementary Figure 1). Altogether, 14 cases with *POLE* and *POLQ* mutations (*E/Q*), 16 cases with *POLE* and *POLZ/REV3L* mutations (*E/Z*) and 20 cases with *POLE, POLQ* and *POLZ/REV3L* (*E/Q/Z*) mutations were identified in the PANCAN cohort (Fig. 1c). Mutations in the exonuclease domain of *POLE* are responsible for causing the ultramutator phenotype in colorectal and uterine corpus cancers [1, 2, 11, 41]. In order to determine the contribution of POLQ and REV3L to the ultramutator phenotype, we compared the mutation frequencies of tumors with mutations in only *POLE to E/Q, E/Z and E/Q/Z* mutant tumors. Mutation frequencies in the cellular genome increased in the following order: no *POLE* mutations < *POLE-*only mutations (anywhere with the POLE exome) < *E/Q*, *E/Z*, *E/Q/Z* mutations (Fig. 1d). The median mutation count of *E/Q/Z* tumors was more than 10-fold higher (*p* < 0.001) than that of tumors with only *POLE* mutations. E + Q and E + Z mutant tumors also displayed significantly higher mutation counts compared to E-only mutant tumors, suggesting a contribution of mutationally altered *POLQ* or *POLZ/REV3L* to the overall cancer mutation rates. Next, we determined the number of mutations in the exonuclease and polymerase domains of *POLE* in the 15 cancer types within our PANCAN compendium (Fig. 1e). The percentage of *POLE* mutations in the exonuclease domain was greater in *E/Q*, *E/Z* and *E/Q/Z* mutant tumors compared to *POLE-only* mutant tumors (*p* = 0.013). In contrast, the percentage of mutations in the DNA polymerase domain was similar. Thus, our data confirm the notion that mutations in the exonuclease domain of POLE are responsible for ultra-high mutation rates. In addition, mutations in POLQ and REV3L may further increase the mutation burden. However, why mutations in POLQ and REV3L preferentially increase tumor mutation frequencies remains elusive.

**Fig. 2:**
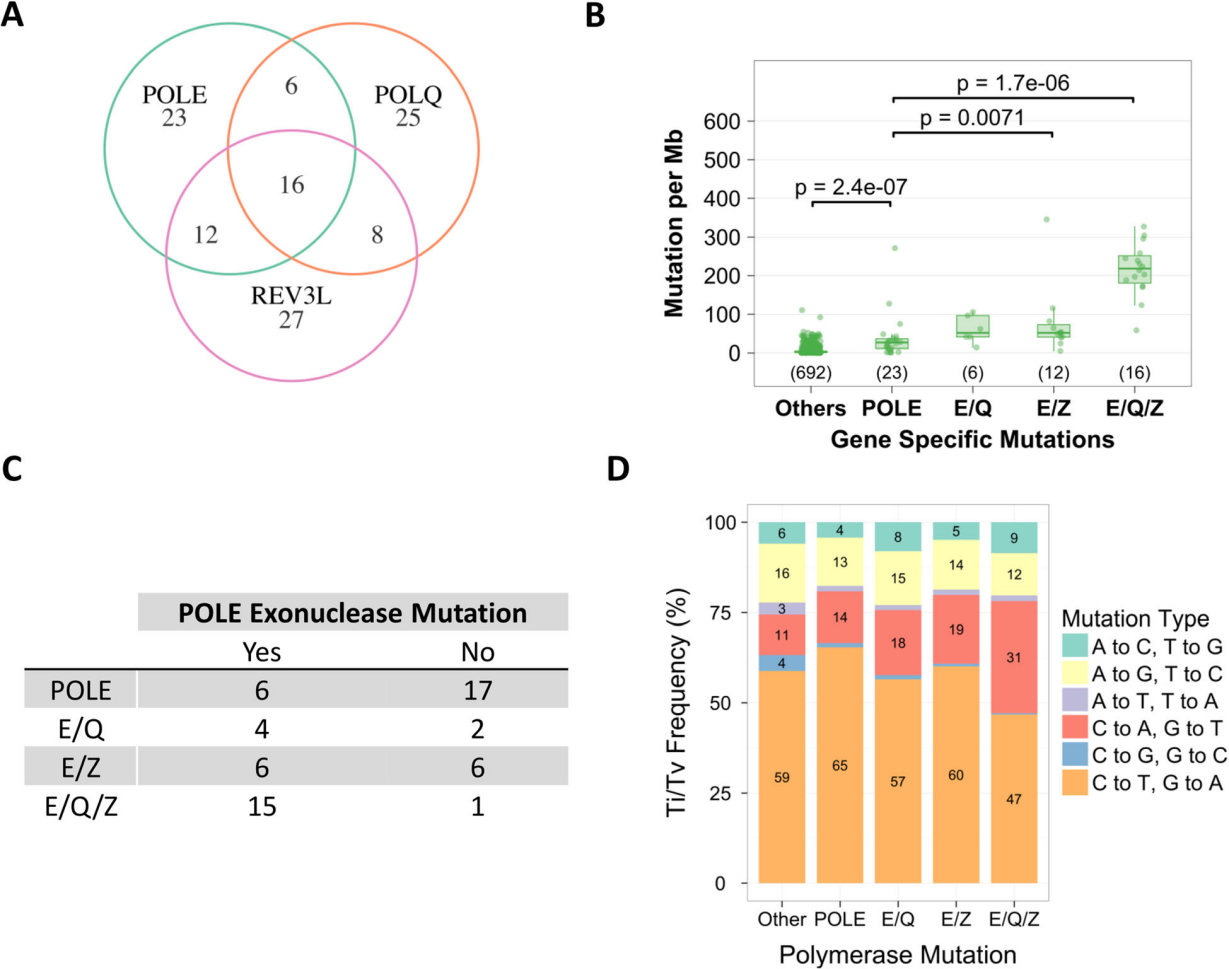
*POLE/Q/Z* mutations *in colorectal* [38]*, endometrial (UCEC) and stomach (STAD) cancers.* **a** Venn diagram displaying the number of cases with mutations in 1, 2 or 3 POL genes. **b** Mutation groups of cases without polymerase mutations (other), or with mutations in *POLE* only, *E/Q*, *E/Z* and *E/Q/Z*. The number of cases in each group is listed in parenthesis. The *p*-value for comparison of POLE and E/Q groups is not significant (*p* = 0.056) **c** Number of cases with mutations in *POLE* exonuclease domain in various mutation groups. **d** Percentages the ratio of transitions (Ti) and transversions (Tv) are shown on the Y-axis for CORE, STAD and UCEC. The x-axis shows the mutation groups

To further investigate a potential role of these mutant DNA polymerases in the ultramutator phenotype, we focused on colorectal [38], endometrial (UCEC) and stomach (STAD) cancers. These cancer types contain the highest numbers of tumors with *E/Q*, *E/Z* and *E/Q/Z* amongst the 15 cancer types included in PANCAN (Fig. 1a, Supplementary Table 1). Among these 3 cancer types, we identified 6 cases with *E/Q*, 12 cases with *E/Z* and 16 cases with *E/Q/Z* mutations (Fig. 2a). In these cancers, the mutation burden in *POLE*, *E/Q*, *E/Z* and *E/Q/Z* mutant tumors paralleled the mutation burden in the whole PANCAN cohort (compare Fig. 2b and Fig. 1c, Supplementary Figure 2A-D). *E/Q/Z* mutant tumors demonstrated, on average, an 8-fold increase in mutation frequencies compared to tumors with only *POLE* mutations. Amongst *POLE* mutant tumors, 26 tumors carried mutations outside the POLE exonuclease domain, while 31 tumors carried mutations within the exonuclease domain. The frequency of POLE exonuclease mutations (15/16 cases in Fig. 2c) provides a valid explanation for the difference in mutation rates and potential association of POLE exonuclease domain mutations with mutations in POLQ and POLZ/REV3L, which are the other two most frequently mutated DNA polymerases.

Overall, exonuclease domain mutations were identified in 6/23 cases of *POLE* only mutant tumors, 4/6 *E/Q* cases, 6/12 *E/Z* cases and 15/16 of *E/Q/Z* cases (Fig. 2c). Stratified by cancer types, *POLE* exonuclease domain mutations occurred in 7/15 colorectal, 18/26 endometrial and 6/16 stomach tumors, demonstrating cancer type specific frequencies (Supplementary Figure 3A). In contrast to the *POLE* gene that demonstrates mutational hotspots in the exonuclease domain, mutational hotspots in the *POLQ* gene are not associated with a functional protein domain (Supplementary Figure 3B, C & D)*.* While *REV3L* does not reveal mutational hotspots, approximately 50% of mutations lead to truncated protein expression (Supplementary Figure 3E & F). Another characteristic of *POLE* mutant tumors are C to A and G to T transversions [2]. We observed the greatest increase of nucleotide transversions in cancers with *E/Q/Z* mutations (Fig. 2d), consistent with the loss of *POLE* exonuclease activity in these tumors.

**Fig. 3:**
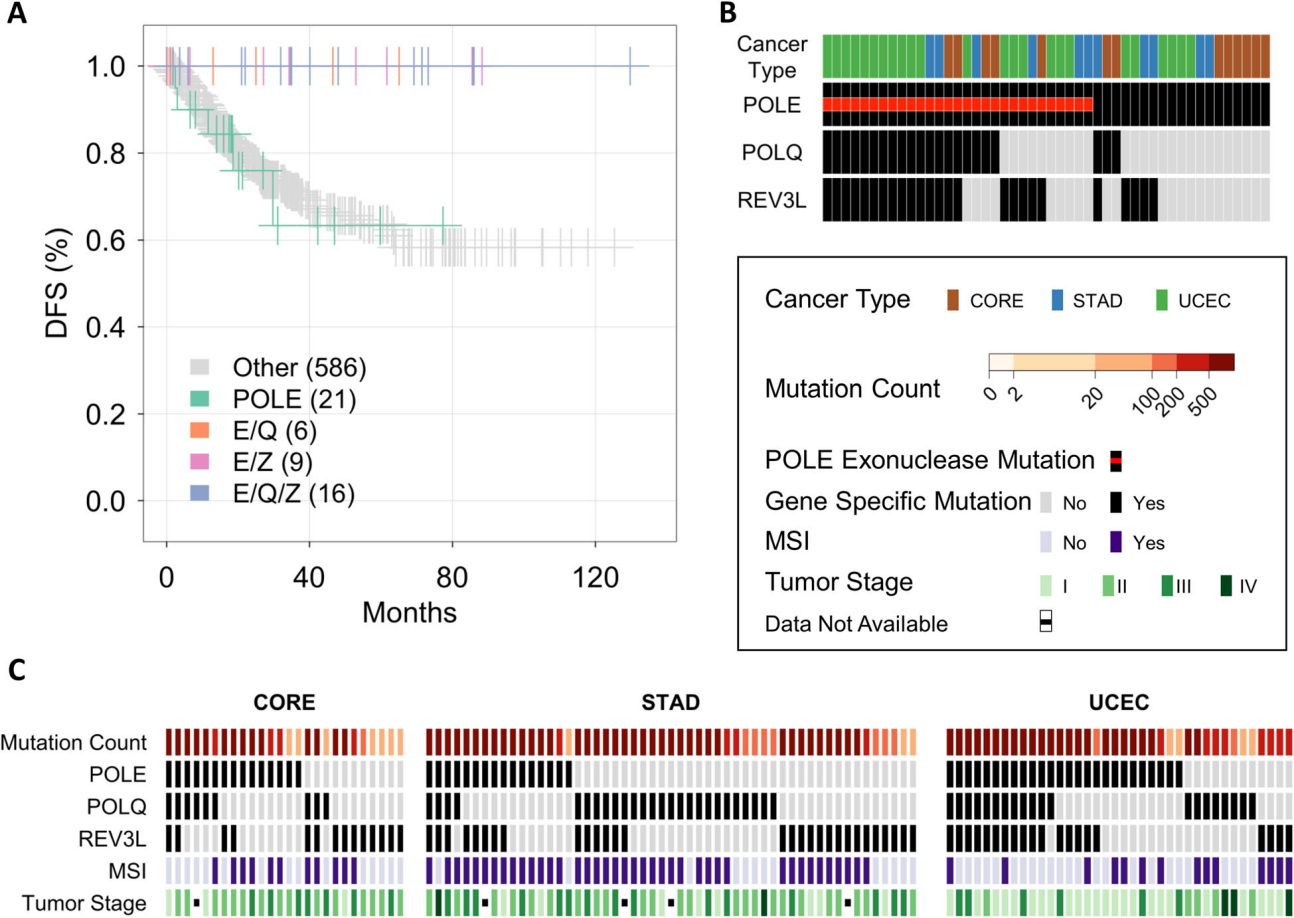
*Survival and clinical characteristics of patients with polymerase mutations colorectal* [38]*, endometrial (UCEC) and stomach (STAD) cancers.* **a** Kaplan-Meier curves of Disease-Free Survival (DFS) for 3 groups of patients: POLE only (*n* = 21, median follow-up =18.4 months), green line; E/Q (*n* = 6, median follow-up = 19.0 months), orange line; E/Z (*n* = 9, median follow-up = 34.3 months), pink line, and E/Q/Z (*n* = 16, median follow-up = 37.5 months), blue line; tumors without mutations in *POLE*, *POLQ* or *REV3L* exomes, grey line. The overall p-value is *p* = 0.0477. Individual *p*-values: E/Q/Z vrs. POLE – *p* = 0.021; E/Q/Z vrs. None – *p* = 0.018, E/Q or E/Z or E/Q/Z vrs. POLE – *p* = 0.003. **b** Polymerase mutation analysis of cases in the good survival group in panel A. The red bar indicates cases with POLE exonuclease mutations. **c** Cancer type-specific illustration of mutation count, *POLE*, *POLQ* and *REV3L* mutations, microsatellite instability (MSI) and tumor stage

Since mutations in *POLE* confer increased disease free survival (DFS) in patients with uterine cancer, even in those patients with high-grade tumors [3, 42], we investigated the prognostic role of *POLQ* and *REV3L* mutations in *POLE* mutant tumors. Kaplan-Meier curves were constructed for colorectal, endometrial and stomach cancer cases with follow-up data (Fig. 3a). Using the TCGA annotations of DFS in individual patients, no cancer recurrences were observed in the E/Q, *E/Z* and *E/Q/Z* mutant groups. POLE exonuclease domain mutations were observed in 29 cases in the good survival group and 1 case in the poor survival group, consistent with the expected long DFS periods of patients with POLE exonuclease domain positive tumors. In addition, 19 cases with mutations in POLE outside the exonuclease domain were in the good survival group. Of those 7 (37%) had concurrent mutations in POLQ or REV3L or in both polymerases (Fig. 3b). Furthermore, a Kaplan-Meier analysis in the PANCAN cohort revealed improved DFS associated with mutations in POLQ and REV3L. The favorable survival outcome was observed in colorectal, endometrial, and stomach cancers. However, no favorable outcome was observed in diffuse B-cell lymphoma (*p* = 0.35). These data provide preliminary evidence of cancer-type specific, favorable survival outcomes in tumors with *POLE* mutations that are located outside the *POLE* exonuclease domain if concurrent mutations in *POLQ*, *REV3L* or in both polymerases are present.

Compared to microsatellite stable tumors (MSS), microsatellite instability (MSI) in colorectal cancer confers a better prognosis [43]. To determine whether the favorable outcome of *E/Q*, *E/Z* and *E/Q/Z* mutant cancers can be explained by MSI or TMN stage, we examined the relationship between MSI status, tumor stage and polymerase mutations in colorectal, endometrial and stomach cancers (Fig. 3c and Supplementary Figure 4 and Supplementary Table 2). Despite improved DFS rates, the full range of tumor stages was observed amongst *E/Q*, *E/Z* and *E/Q/Z* tumors (*p* = 0.42) (Supplementary Table 2A). Comparing the POLE-only and E/Q/Z mutant cancers did not reveal a significant difference in tumor stage, but differed in the frequency of MSI cases (*p* < 0.001). POLE mutant tumors were more frequent in the MSI group (29/163, 17.8%) than in the MSS group (30/595, 5%). In addition, the frequency of MSI cases in *POLE* mutant tumors differed between the 3 cancer types (p < 0.001) (Supplementary Table 2B & C). Although MSI is enriched in samples with high mutation levels (Supplementary Table 2D), as expected, 10-fold higher mutation counts (*P* < 0.001) were observed in cancers with *E/Q/Z* mutations compared to MSI without E/Q/Z mutations (Supplementary Figure 5). These results suggest that mutations in *E/Q*, *E/Z* and *E/Q/Z* confer a better prognosis independent of MSI status and TMN stage in colorectal, endometrial and stomach adenocarcinomas.

**Fig. 4:**
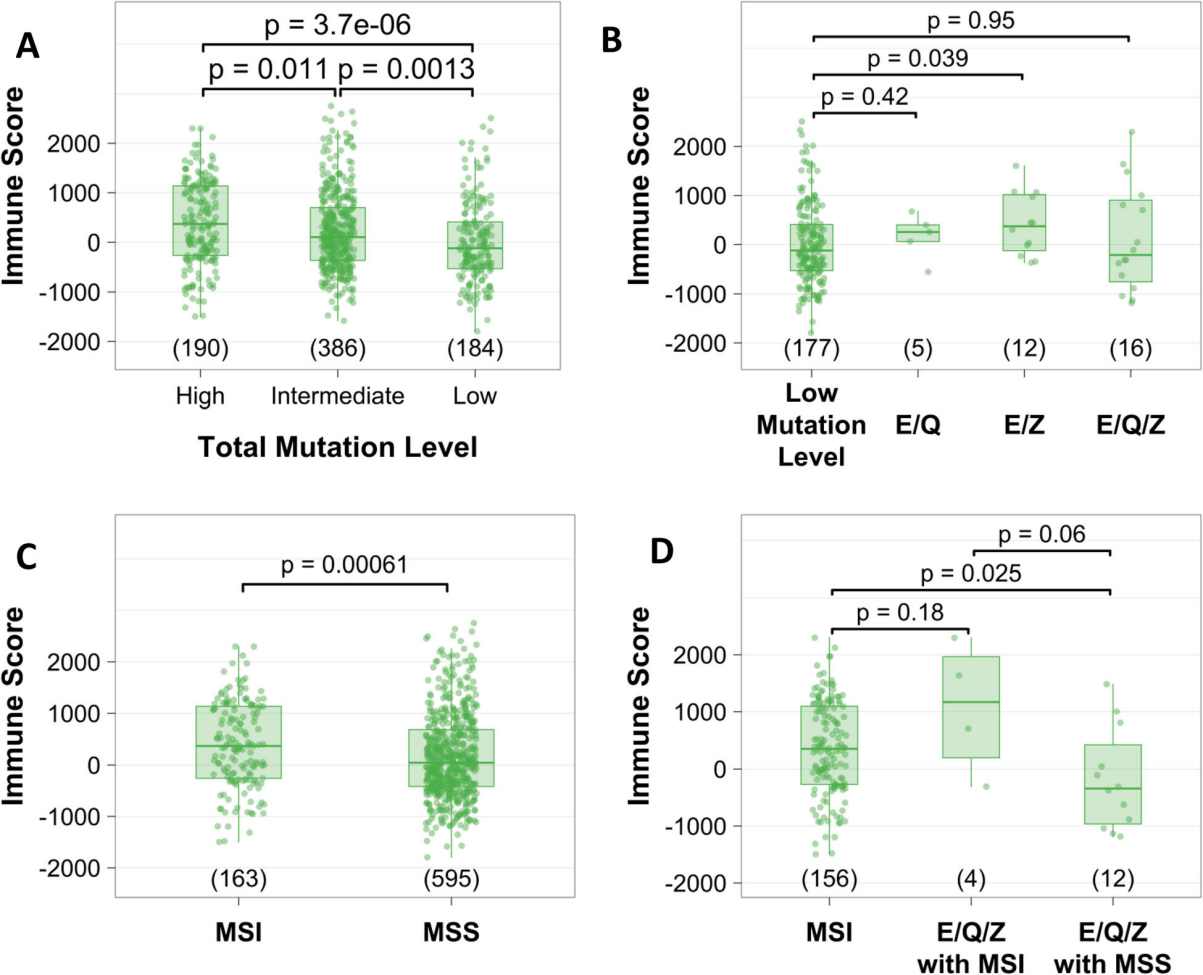
*ESTIMATE immune scores by mutation frequency quartiles, E/Q/Z mutation groups, and MSI in colorectal* [38]*, endometrial (UCEC) and stomach (STAD) cancers*. **a** ESTIMATE immune scores in cancers within high, intermediate and low overall mutation quartiles. **b** Immune scores of samples with *E/Q*, *E/Z* and *E/Q/Z* mutations compared to the low mutation quartile from panel A. **c** Immune scores in groups of cancers separated by MSI status. **d** Immune scores in MSI and MSS *E/Q/Z* cases compared to all other MSI cases. For each panel the number of cases within each group is included in parentheses on the x-axis

We next examined the amount of the cancer-associated immune infiltrate. The immune score obtained through ESTIMATE [37] corresponded to the categorical score of the immune infiltrate derived from digital H&E images (Supplementary Fiure 6). Therefore, we used the ESTIMATE immune scores for further analysis of colorectal, endometrial and stomach cancers. As shown in Fig. 4a, a significant difference was observed in the median immune scores between groups with low, intermediate and high mutation burden, grouped based on mutation burden and not on E, Q, Z mutant status (see Methods) and, as expected, the median immune scores increased with total mutation levels. Surprisingly, the immune scores in *E/Q/Z* mutant tumors did not differ significantly from tumors with a low level of mutations (Fig. 4b). As expected, MSI tumors possessed higher immune scores than MSS tumors (*p* < 0.001) [44] (Fig. 4c). Finally, immune scores of MSI and *E/Q/Z* mutation tumors were similar to those in the MSI group and higher than MSS and *E/Q/Z* mutation tumors (Fig. 4d). Within the group of tumors with *POLE* exonuclease domain mutations, MSS tumors possessed lower immune scores than MSI tumors, but the difference was not significant (*p* = 0.29) (Supplementary Figure 7). Together, results in this TCGA cohort demonstrate that the immune response is driven by MSI rather than POLE exonuclease domain mutations.

## Discussion

An analysis of 14 DNA polymerases in tumors with mutations in the *POLE* revealed additional mutations in specific polymerases, most commonly in *POLQ* and *POLZ/REV3L*. Among the 15 cancer types, colorectal, uterine and stomach cancer were most frequently afflicted by these mutations. Cancers with mutations in *POLE* and *POLQ* (*E/Q*), *POLE* and *POLZ/REV3L (E/Z)* and in all 3 polymerases (*E/Q/Z*) were associated with the highest mutation burden and an excellent prognosis independent of MSI status and tumor stage. Mutations in the exonuclease domain were observed in 94% (15/16) of E/Q/Z mutant tumors, but only in 26% of *POLE*-only mutant tumors or in 55% of E/Q + E/Z tumors. However, despite harboring 10-fold more mutations than MSI tumors and 8-fold more mutations than the mutation frequencies associated with *POLE*-only mutant tumors, *E/Q/Z* mutant tumors did not display significantly more inflammation.

The main result from that analysis is that patients with colorectal, stomach and endometrial cancers bearing *E/Q*, *E/Z* and *E/Q/Z* mutations have 100% disease free survival (DFS) at a median follow up time of 33 months. In contrast, patients with tumors bearing mutations in *POLE* only, most of which outside the *POLE* exonuclease domain, had a DFS of 76% at follow up of 18.4 months. The favorable DFS in *E/Q*, *E/Z* and *E/Q/Z* mutated tumors occurred even in tumors with mutations in POLE that are located outside the POLE exonuclease domain. The contribution of mutations in POLE to TMB, C➔A substitutions and cancer type associations are described in Table 1 of Raynor et al., 2016 using a larger resource of cases and should be used to interpret the mutations in the current study, listed in Supplementary Figure 3. As a whole, the current study expands the spectrum of *POLE* mutant tumors with an excellent prognosis. The favorable prognosis included patients with high tumor stage, which echoes prior studies demonstrating a favorable outcome of uterine tumors with *POLE* exonuclease mutations despite adverse standard clinicopathologic indicators including high grade, high stage, and lymphovascular invasion [3, 45]. While the high mutation frequencies may cause an early growth advantage [46], as tumors evolve they may succumb to high mutation burden as new mutations can no longer be tolerated and cause tumor cell death [47, 48] or increased sensitivity to therapeutic agents.

The prevailing hypothesis for the favorable prognosis of cancers displaying the hypermutator phenotype is the increased attack by the immune system. Evidence in support of this theory is the observation that tumor infiltrating [49] and peritumoral lymphocytes are increased and that cytotoxic activities in CD8+ and CD4+ lymphocyte populations are heightened in *POLE* mutated endometrial cancers [50–53], similar to hypermutated MSI tumors [54]. This observation has led to the hypothesis that immune checkpoint inhibitors may be efficacious in *POLE* ultramutated tumors [51]. Our results question a direct relationship between mutation burden, tumor immune response and PD-L1 expression, also raised in a larger study across 5722 cases from 21 cancer types in TCGA [55]. While we observed a concordance between the computational and histological assessments of the immune infiltrate, the immune score in tumors with *E/Q/Z* mutations depended on MSI status. This result suggests that the immune infiltrate attributable to mutations in *E/Q/Z* mutant tumors may be less, or that its composition may involve immune cells other than lymphocytes. Lesser CD8+ and gamma-interferon gene expression signatures have also been observed in gastrointestinal tumors with a large single nucleotide variant (SNV) burden that was attributed largely to POLE exonuclease mutations [49]. Perhaps E/Q/Z mutations occur at a later point in tumor evolution [56] when immunosuppressive factors already dominate. We also cannot rule out the possibility of increased numbers of cytotoxic lymphocytes intermixed with E/Q/Z mutant tumor cells, because computational methods and inspection of H&E images are not sensitive enough to detect small differences in tumor infiltrating lymphocytes (TILs) that may have large anti-tumoral effects.

A significant limitation of the study lies in the relatively small number of tumors. This limitation cautions the generalization of results and seemingly novel insights into the hypermutator phenotype. Studies by other groups attributed hypermutator phenotype to specific mutations primarily within the *POLE* exonuclease domain. Given the proofreading function of the exonuclease domain, it makes sense that mutations outside the domain have a lesser effect on TMB. In agreement with this concept, our study reveals that (1) compared to tumors harboring only a mutation in *POLE* (*POLE* single-mutant tumors, 6 of 23 cases), *POLE* exonuclease domain mutations are more common in tumors with both double (*E/Z* and *E/Q*) and triple (*E/Q/Z*) DNA polymerase mutations (25 of 34 cases) and (2) double and triple mutant tumors have higher mutation counts than *POLE* single mutant tumors. While it cannot be fully excluded that POLQ and POLZ/REV3L mutations are bystander events in POLE mutant tumors, we observe a higher TMB in cases with mutations in all three polymerases (Supplementary Figure 2A and B). Mechanistically, POLQ and POLZ are thought to function in different repair processes: POLQ in alternative (microhomology-mediated) non-homologous DNA repair pathway and POLZ in translesion DNA synthesis. How these DNA repair processes cooperate with the replicative DNA polymerase, POLE, to prevent genome instability remains unknown. This will be an important subject for further understanding of the mechanism underlying the hypermutator phenotype.

## Conclusions

If validated in additional cohorts, our findings may have important clinical implications. They build upon and expand the previously well documented good prognostic impact of *POLE* exonuclease mutations in uterine cancer, that have generated intense interest in part due to the paradox of a favorable prognosis in tumors with pathologic indicators of poor prognosis. While in this study, prolonged DFS is observed in colorectal, endometrial and stomach cancers with *E/Q/Z* mutations, this is not the case in other non-carcinoma cancer types within TCGA. Thus, we find that the positive outcome prediction is cancer type specific. Altogether, results from this study provide a rationale for including POLQ and/or POLZ/REV3L mutations in clinical outcome studies of tumors with POLE mutations. However, future validation is required to confirm the concept that is revealed in the current study.

## Supplementary information

**Additional file 1: Supplementary Figure 1.** Number of cases POLE mutations (*n* = 138) and mutations in the exomes of DNA polymerase genes in PANCAN. **Supplementary Figure 2.** Mutation counts for colorectal (CORE), stomach (STAD) and endometrial (UCEC) cancers by specific polymerase mutated groups in TCGA data sets. **Supplementary Figure 3.** Locations of mutations in POLE, POLQ and REV3L exomes in individual colorectal (CORE), stomach (STAD) and uterine cancers (UCEC). **Supplementary Figure 4.** Relationships between mutation spectrum and mutation counts, POLE, POLQ and REV3L exome mutations, MSI and tumor stage of individual cases. **Supplementary Figure 5.** Mutation rates per Mb (y-axis) of CORE, STAD and UCEC cases with MSI and E/Q, E/Z and E/Q/Z (x-axis) mutations. **Supplementary Figure 6.** Relationship between pathology inflammation score and ESTIMATE immune scores for CORE, STAD, and UCEC. **Supplementary Figure 7.** ESTIMATE immune scores in colorectal (CORE), endometrial (UCEC) and stomach (STAD) cancers. **Supplementary Table 1.** Number of cases with POLE, POLQ, Z/REV3L, or multiple exome mutations in the PANCAN cohort. **Supplementary Table 2.** Contingency tables showing number of cases of colorectal, endometrial and stomach cancers in each category.

## Data Availability

Sequencing data can be obtained from National Cancer Institue GDC data portal and are published in [57]. Raw genomic and clinical data can be found at the NCI Genomic Data Commons https://portal.gdc.cancer.gov/legacy-archive/ The MC3 mutation annotation file can be accessed at https://gdc.cancer.gov/about-data/publications/mc3-2017 and the processed data files can be viewed at https://gdc.cancer.gov/about-data/publications/pancanatlas

## Abbreviations

POLE: Polymerase epsilon
POLQ: Polymerase theta
POLZ/REV3L: REV3 like, DNA directed polymerase zeta (POLZ) catalytic subunit
TCGA: The Cancer Genome Atlas
MSI: Micro Satellite Instability
MSS: Micro Satellite Stability
TMB: Tumor Mutation Burden
TNM: Tumor, lymph Node, Metastasis
ESTIMATE: Estimation of STromal and Immune cells in MAlignant Tumor tissues using Expression data
