# Supplementary material for: Mutant POLQ and POLZ/REV3L DNA polymerases may contribute to the favorable survival of patients with tumors with POLE mutations outside the exonuclease domain
Source: BMC Med Genet. 2020 Aug 24;21:167. doi: 10.1186/s12881-020-01089-9 (PMC7446057; doi:10.1186/s12881-020-01089-9)
Supplement: Supplementary file 1 — Additional file 1: Supplementary Figure 1. Number of cases POLE mutations (n = 138) and mutations in the exomes of DNA polymerase genes in PANCAN. Supplementary Figure 2. Mutation counts for colorectal (CORE), stomach (STAD) and endometrial (UCEC) cancers by specific polymerase mutated groups in TCGA data sets. Supplementary Figure 3. Locations of mutations in POLE, POLQ and REV3L exomes in individual colorectal (CORE), stomach (STAD) and uterine cancers (UCEC). Supplementary Figure 4. Relationships between mutation spectrum and mutation counts, POLE, POLQ and REV3L exome mutations, MSI and tumor stage of individual cases. Supplementary Figure 5. Mutation rates per Mb (y-axis) of CORE, STAD and UCEC cases with MSI and E/Q, E/Z and E/Q/Z (x-axis) mutations. Supplementary Figure 6. Relationship between pathology inflammation score and ESTIMATE immune scores for CORE, STAD, and UCEC. Supplementary Figure 7. ESTIMATE immune scores in colorectal (CORE), endometrial (UCEC) and stomach (STAD) cancers. Supplementary Table 1. Number of cases with POLE, POLQ, Z/REV3L, or multiple exome mutations in the PANCAN cohort. Supplementary Table 2. Contingency tables showing number of cases of colorectal, endometrial and stomach cancers in each category. [file 12881_2020_1089_MOESM1_ESM.pdf]

SUPPLEMENTARY FILES.

A novel stratification of tumors with POLE mutations by mutations in additional DNA polymerases

Fangjin Huang[1], Hisashi Tanaka[2], Beatrice S. Knudsen[1,3]* and Joanne K. Rutgers[3]*

[1]Department of Biomedical Sciences, [2]Surgery and [3]Pathology and Laboratory Medicine, Cedars-Sinai Medical Center, Los Angeles, CA 90048

*equal contribution

Supplementary Figure 1

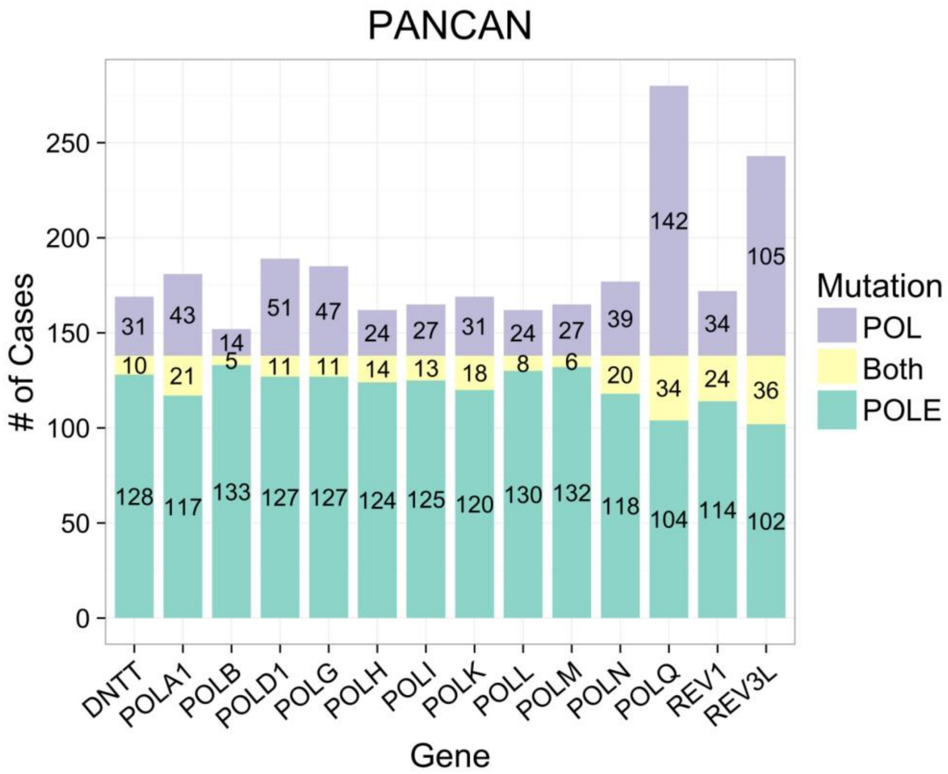

Supplementary Figure 1. Number of cases POLE mutations (n=138) and mutations in the exomes of DNA polymerase genes in PANCAN. Each case was analyzed for mutations in the 14 DNA polymerases listed on the x-axis. Numbers indicate cases with mutations in *POLE* (green), the other DNA polymerase (purple) and cases with mutations both polymerases (yellow).

Supplementary Figure 2

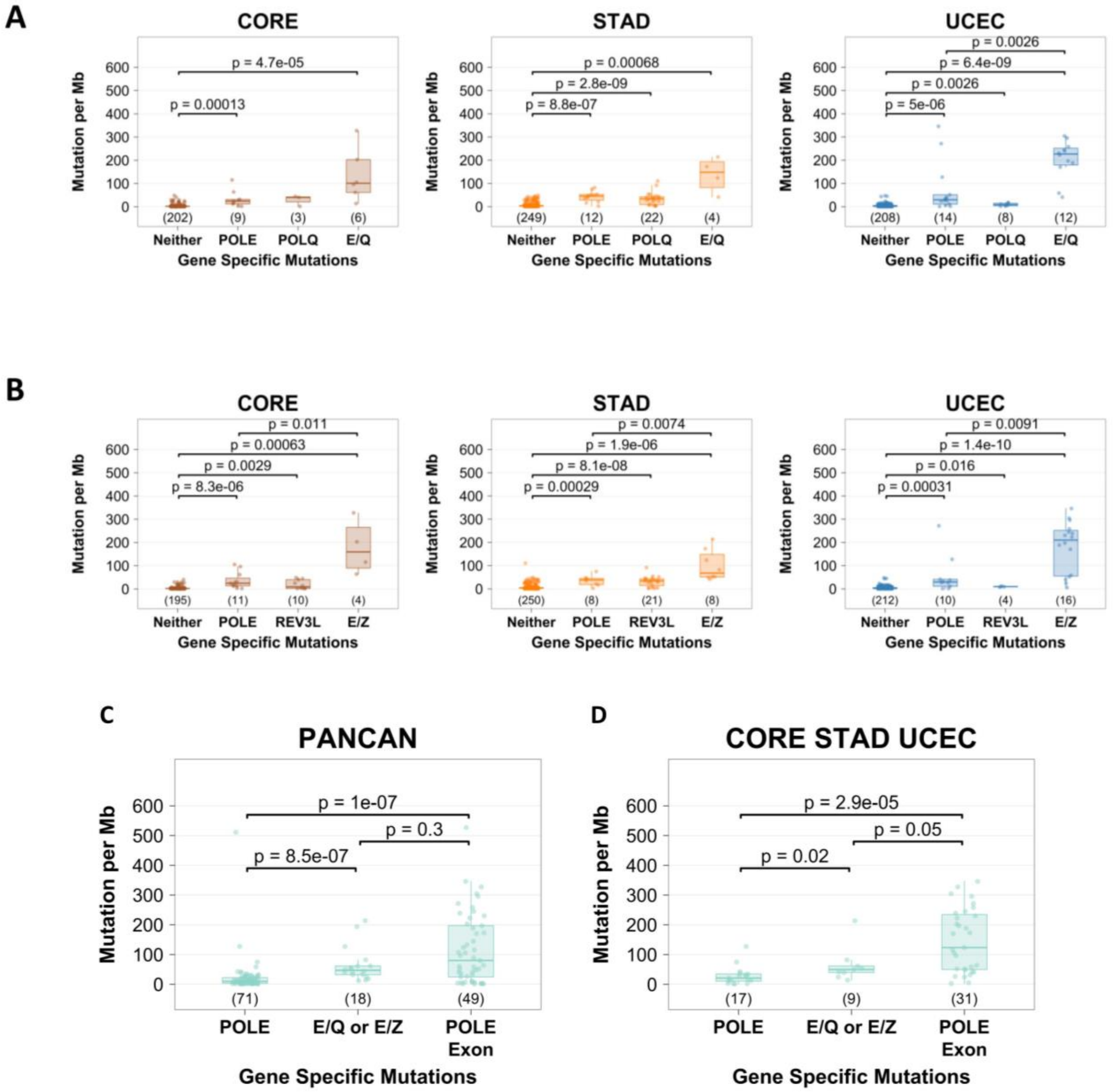

Supplementary Figure 2. Mutation counts for colorectal (CORE), stomach (STAD) and endometrial (UCEC) cancers by specific polymerase mutated groups in TCGA data sets. A) Mutation count of cases with POLE and/or POLQ mutations. The first bar illustrates cases without mutations in the exome of POLE or POLQ, the second and third bars show cases with mutations in POLE or POLQ exomes, respectively, and the fourth bar depicts cases in which both, POLE and POLQ are mutated. The y-axis shows the mutation count per megabase (Mb). **B)** Same as panel A for POLZ/REV3L. **C) and D).** Mutation counts in tumors with POLE exonuclease domain mutations compared to tumors with POLE non-exonuclease domain mutations and tumors with POLE non-exonuclease domain mutations, but mutations in POLQ or REV3L. Panel C shows the results of the analysis in PANCAN, while panel D shows the results of the analysis in CORE, STAD and UCEC tumors.

Supplementary Figure 3 A,B

A

Cancer.Study	Sample.ID	Mutation.Count	DNA_pol_B_exo1	DNA_pol_B	DUF1744	POLE_Other
CORE	TCGA-AG-A002-01	9822	1	0	0	1
	TCGA-AA-A010-01	6086	1	0	0	1
	TCGA-AA-A00N-01	3469	1	0	0	1
	TCGA-AA-3984-01	3165	1	0	0	0
	TCGA-AA-3977-01	2896	1	1	0	0
	TCGA-AA-3864-01	1920	1	0	0	0
	TCGA-AG-3892-01	1851	0	0	0	1
	TCGA-AA-3710-01	952	0	0	0	1
	TCGA-AA-A00J-01	864	0	0	0	1
	TCGA-AA-3555-01	735	1	0	0	0
	TCGA-AA-3525-01	465	0	2	0	1
	TCGA-AA-3518-01	424	0	0	0	1
	TCGA-AA-3833-01	375	0	0	0	1
	TCGA-AG-A01W-01	96	0	0	0	1
	TCGA-AA-3678-01	58	0	0	1	0
STAD	TCGA-BR-6452-01	6415	0	2	1	1
	TCGA-BR-8680-01	5189	1	0	0	1
	TCGA-CG-5721-01	3705	1	2	0	0
	TCGA-BR-4361-01	2457	0	0	1	0
	TCGA-CD-A4MG-01	2239	0	0	1	0
	TCGA-HU-A4GQ-01	1608	0	0	1	0
	TCGA-BR-4362-01	1547	1	0	0	0
	TCGA-HU-A4GT-01	1493	0	0	0	1
	TCGA-BR-8361-01	1438	1	1	0	0
	TCGA-HU-A4GU-01	1275	0	0	0	1
	TCGA-CG-5726-01	1242	0	0	0	1
	TCGA-CG-5728-01	1241	1	0	0	0
	TCGA-HU-A4H8-01	1047	0	1	0	0
	TCGA-BR-8059-01	629	0	0	0	2
	TCGA-F1-A448-01	485	0	0	0	1
	TCGA-HF-7136-01	75	1	0	0	0
UCEC	TCGA-AP-A0LM-01	10379	2	0	0	0
	TCGA-AP-A059-01	9130	1	0	0	3
	TCGA-B5-A0JY-01	8871	1	1	0	0
	TCGA-B5-A11E-01	8146	1	0	1	1
	TCGA-B5-A0UV-01	7749	1	0	1	1
	TCGA-B5-A0UF-01	7354	1	0	0	1
	TCGA-AP-A056-01	7170	1	0	1	1
	TCGA-AX-A0J0-01	6907	1	0	0	0
	TCGA-AP-A051-01	6684	1	0	0	0
	TCGA-D1-A103-01	5911	1	1	1	2
	TCGA-AX-A05Z-01	5654	1	0	1	1
	TCGA-D1-A17Q-01	5113	1	0	0	0
	TCGA-AX-A0J1-01	3817	0	0	0	1
	TCGA-D1-A16X-01	1751	1	1	0	0
	TCGA-B5-A11N-01	1550	1	0	0	0
	TCGA-A5-A0GP-01	1234	1	0	0	0
	TCGA-B5-A0TC-01	1193	0	0	0	1
	TCGA-A5-A0VP-01	1130	0	1	0	0
	TCGA-D1-A16Y-01	948	1	0	0	0
	TCGA-AP-A054-01	829	0	0	1	0
	TCGA-B5-A11Y-01	799	1	0	0	1
	TCGA-B5-A11H-01	724	0	0	0	1
	TCGA-BG-A0LX-01	321	0	0	1	0
	TCGA-BG-A0VX-01	160	1	0	0	0
	TCGA-BG-A18A-01	51	0	1	0	0
	TCGA-AP-A1DQ-01	26	0	0	0	1

POLE

B

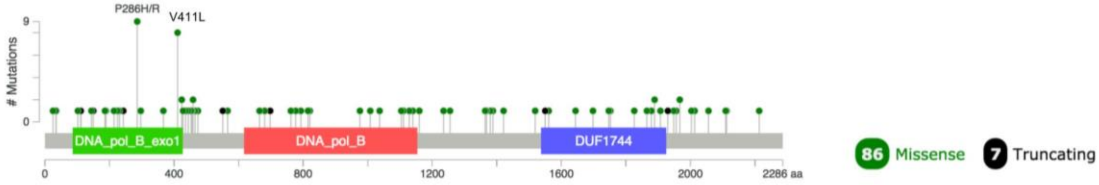

Supplementary Figure 3 C,D

C

Cancer.Study	Sample.ID	Mutation.Count	DEAD	Helicase_C	DNA_pol_A	POLQ_Othe
COAD	TCGA-AG-A002-01	9822	0	1	0	2
	TCGA-AA-A010-01	6086	0	0	0	1
	TCGA-AA-3984-01	3165	0	0	0	1
	TCGA-AA-3977-01	2896	0	0	0	1
	TCGA-AG-3892-01	1851	0	0	0	1
	TCGA-AA-3672-01	1343	0	0	0	2
	TCGA-AA-3947-01	1208	1	0	0	0
	TCGA-AA-3518-01	424	0	0	1	0
	TCGA-AA-3971-01	59	0	0	0	1
STAD	TCGA-BR-6452-01	6415	0	0	1	1
	TCGA-BR-8680-01	5189	0	1	0	1
	TCGA-CG-5721-01	3705	1	0	0	0
	TCGA-BR-4184-01	3313	0	0	0	1
	TCGA-BR-8487-01	2760	0	0	1	0
	TCGA-BR-A4QL-01	1633	1	0	0	0
	TCGA-CG-5723-01	1558	0	0	1	0
	TCGA-CG-4442-01	1385	0	0	0	1
	TCGA-BR-7851-01	1284	1	0	0	0
	TCGA-BR-7707-01	1268	0	0	0	1
	TCGA-CG-5728-01	1241	0	0	0	1
	TCGA-BR-4201-01	1212	0	1	0	0
	TCGA-B7-5816-01	1168	0	1	0	0
	TCGA-HF-7132-01	1093	0	0	1	0
	TCGA-CG-5733-01	1042	0	0	0	1
	TCGA-BR-8589-01	968	0	1	0	0
	TCGA-BR-4257-01	892	0	1	0	0
	TCGA-F1-6874-01	838	0	0	0	1
	TCGA-BR-8382-01	744	0	0	0	1
	TCGA-BR-7703-01	650	0	0	0	1
	TCGA-BR-8284-01	268	0	0	0	1
	TCGA-FP-7829-01	207	0	0	1	0
	TCGA-BR-7716-01	136	0	0	0	1
	TCGA-BR-A4PD-01	130	1	0	0	0
	TCGA-CG-4440-01	110	0	1	0	0
	TCGA-IN-8462-01	99	0	0	0	1
UCEC	TCGA-AP-A059-01	9130	0	0	1	1
	TCGA-B5-A0JY-01	8871	0	0	0	1
	TCGA-BS-A0UV-01	7749	0	0	0	2
	TCGA-BS-A0UF-01	7354	0	0	0	2
	TCGA-AP-A056-01	7170	0	0	1	1
	TCGA-AX-A0J0-01	6907	1	0	1	0
	TCGA-AP-A051-01	6684	1	1	1	0
	TCGA-D1-A103-01	5911	0	0	0	1
	TCGA-AX-A05Z-01	5654	1	0	0	1
	TCGA-D1-A17Q-01	5113	0	0	0	1
	TCGA-D1-A16X-01	1751	0	0	0	1
	TCGA-A5-A0GP-01	1234	0	1	0	0
	TCGA-B5-A11R-01	533	0	0	0	2
	TCGA-B5-A0K9-01	520	0	0	1	0
	TCGA-DI-A0WH-01	351	0	0	0	1
	TCGA-D1-A17M-01	302	1	0	0	0
	TCGA-EY-A1GS-01	211	0	0	1	0
	TCGA-AP-A0LF-01	176	0	0	0	1
	TCGA-D1-A1NU-01	68	0	0	0	1
	TCGA-AP-A05A-01	62	0	0	0	1

D

POLQ

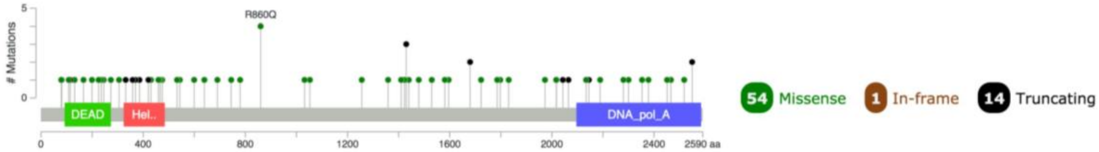

Supplementary Figure 3 E,F

E

Cancer.Study	Sample.ID	Mutation.Count	DNA_pol_B_exo1	DNA_pol_B	zf_C4pol	Other
CORE	TCGA-AG-A002-01	9822	1	0	0	7
	TCGA-AA-A010-01	6086	0	0	0	3
	TCGA-AA-A00N-01	3469	0	0	0	2
	TCGA-AA-3864-01	1920	0	0	0	1
	TCGA-AA-3715-01	1463	0	1	0	0
	TCGA-AA-3672-01	1343	0	0	0	1
	TCGA-AA-3947-01	1208	0	0	0	1
	TCGA-AA-3877-01	759	0	1	0	0
	TCGA-A6-2672-01	416	0	0	0	1
	TCGA-AA-3819-01	134	0	0	0	1
	TCGA-A6-3810-01	95	0	0	0	1
	TCGA-AA-3673-01	65	1	0	0	0
	TCGA-AG-3609-01	62	1	0	0	0
	TCGA-AA-3520-01	54	0	0	0	1
STAD	TCGA-BR-6452-01	6415	1	0	0	3
	TCGA-BR-8680-01	5189	1	0	0	3
	TCGA-CG-5721-01	3705	0	1	1	1
	TCGA-BR-8487-01	2760	0	0	0	1
	TCGA-BR-4361-01	2457	0	0	0	1
	TCGA-BR-A4QL-01	1633	0	0	0	1
	TCGA-HU-A4GQ-01	1608	0	0	0	1
	TCGA-CG-5723-01	1558	0	0	0	1
	TCGA-BR-4362-01	1547	0	0	0	1
	TCGA-HU-A4GT-01	1493	1	1	0	0
	TCGA-HU-8602-01	1373	0	0	0	1
	TCGA-BR-8591-01	1299	0	0	0	1
	TCGA-HU-A4G8-01	1296	0	1	0	0
	TCGA-CG-5726-01	1242	0	0	0	1
	TCGA-F1-6177-01	1171	0	0	0	1
	TCGA-B7-5816-01	1168	0	0	0	1
	TCGA-BR-8363-01	1052	1	0	0	0
	TCGA-CG-5733-01	1042	0	0	0	1
	TCGA-HU-A4GN-01	1024	0	1	0	0
	TCGA-CG-4305-01	1020	0	0	0	1
	TCGA-BR-4257-01	892	0	1	0	0
	TCGA-BR-4256-01	847	0	0	0	1
	TCGA-D7-A4YV-01	788	0	1	0	0
	TCGA-BR-6802-01	416	0	0	0	1
	TCGA-HU-A4H0-01	145	0	0	0	1
	TCGA-BR-8679-01	129	0	0	0	1
	TCGA-HU-A4GC-01	122	0	0	0	1
	TCGA-D7-6524-01	88	0	1	0	0
	TCGA-CD-A489-01	63	0	0	0	1
UCEC	TCGA-AP-A0LM-01	10379	0	0	0	1
	TCGA-AP-A059-01	9130	1	0	0	2
	TCGA-B5-A0JY-01	8871	1	1	1	2
	TCGA-BS-A0UV-01	7749	0	0	0	5
	TCGA-BS-A0UF-01	7354	0	0	0	1
	TCGA-AP-A056-01	7170	0	0	0	1
	TCGA-AX-A0J0-01	6907	1	1	0	0
	TCGA-AP-A051-01	6684	0	0	0	1
	TCGA-D1-A103-01	5911	0	0	0	1
	TCGA-AX-A05Z-01	5654	0	0	0	3
	TCGA-D1-A17Q-01	5113	0	0	0	3
	TCGA-D1-A16X-01	1751	0	0	0	1
	TCGA-B5-A11N-01	1550	0	0	0	1
	TCGA-BS-A0TC-01	1193	1	0	0	0
	TCGA-B5-A11H-01	724	0	0	0	1
	TCGA-D1-A160-01	354	0	0	0	1
	TCGA-BS-A0UL-01	291	0	0	1	0
	TCGA-D1-A176-01	272	0	1	0	0
	TCGA-B5-A11U-01	226	0	0	1	0
	TCGA-BG-A0VX-01	160	0	0	0	1

REV3L

F

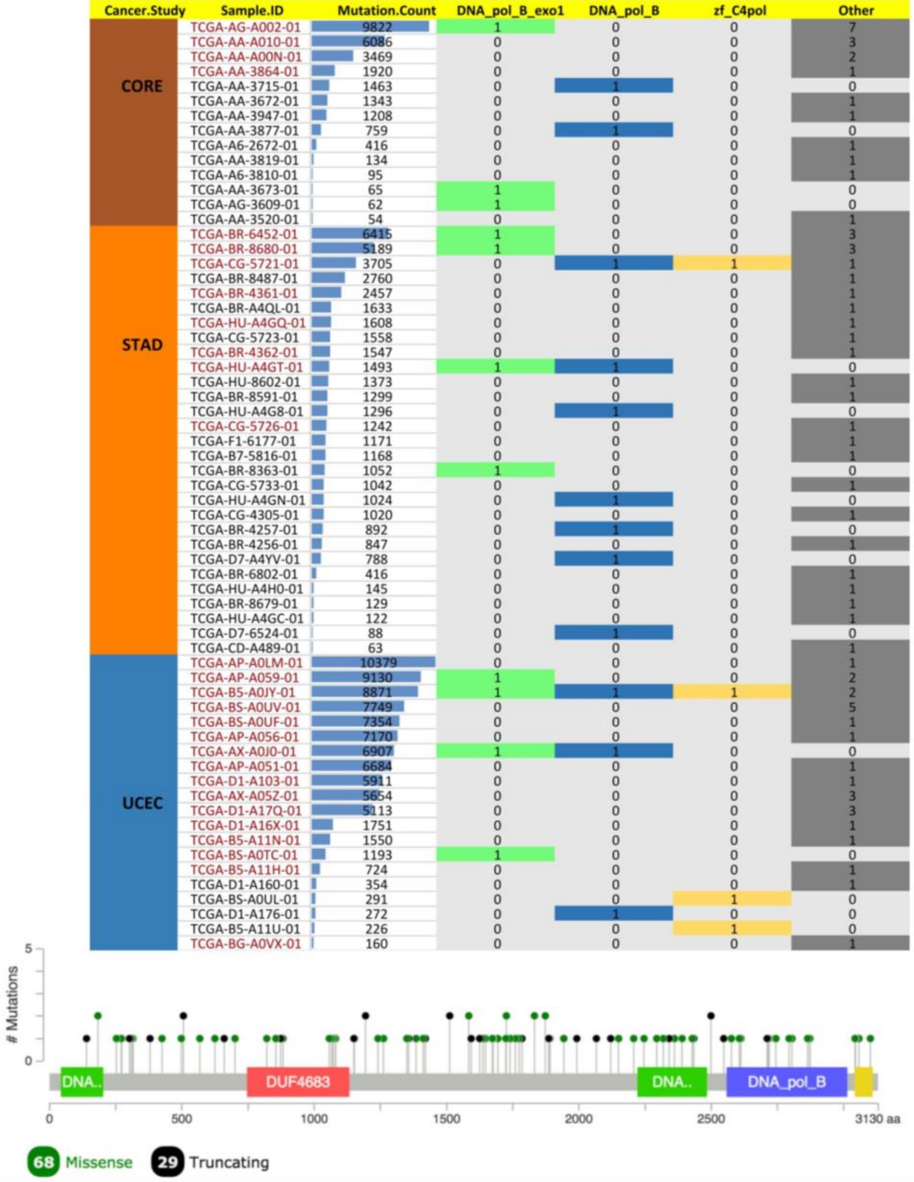

Supplementary Figure 3*. **Locations of mutations in POLE, POLQ and REV3L exomes in individual colorectal (CORE), stomach (STAD) and uterine cancers (UCEC).*** **A) and B)** ***POLE mutations**. **A)*** *Column 1:* Cancer type. *Column 2:* TCGA sample ID for individual tumors: black – POLE mutant, red – E/Q mutant. *Column 3:* Cancers ranked by total mutation count; *Column 4:* green bar denotes mutation(s) in exonuclease domain. *Column 5:* red bar denotes mutation(s) in DNA polymerase domain; *Column 6:* blue bar denotes mutation in domain of unknown function (DUF); *Column 7:* grey bar denotes mutation(s) in other regions of the *POLE* exome. Numbers indicate the total number of mutations per case (Column 3) or per domain (Columns 4-7) **B)** Lollipop plot of mutations in POLE gene. Y-axis shows the number of mutations in a specific nucleotide, black circles illustrate truncating mutations, green circles illustrate missense mutations. Domain colors as described in A.

C) and D) ***POLQ mutations. C)*** *Column 1:* same cancer types as listed in panel A. *Column 2:* TCGA sample ID for individual tumors: black – POLQ mutant, red – E/Q mutant. *Column 3:* same as in panel A. *Column 4:* green bar denotes mutation in DEAD domain. *Column 5:* red bar denotes mutation in Helicase C domain; *Column 6:* blue bar denotes mutation in DNA polymerase domain; *Column 7:* grey bar denotes mutation(s) in other regions of the POLQ exome. Numbers indicate the total number of mutations per case (Column 3) or in the domain of the POLQ gene (Columns 4-7) **D.** Lollipop plot of mutations in POLQ gene. Y-axis shows the number of mutations in a specific nucleotide. Black circles illustrate truncating mutations. Green circles illustrate missense mutations. Brown circles illustrate in-frame deletion. Domain colors as described in C.

E) and F) REV3L mutations in same cancer types listed in panel A. *Column 2:* TCGA sample ID for individual tumors: black – REV3L mutant, red – E/Z mutant. *Column 3:* same as in panel A. *Column 4:* green bar denotes mutation in exonuclease domain. *Column 5:* blue bar denotes mutation in polymerase domain; *Column 6:* yellow bar denotes mutation in the C4-type zinc finger of DNA polymerase delta domain; *Column 7:* grey bar denotes mutation(s) in other regions of the REV3L exome. Numbers indicate the total number of mutations per case (Column 3) or per domain in the REV3L gene (Columns 4-7) **F.** Lollipop plot of mutations in REV3L gene. Y-axis shows the number of mutations in a specific nucleotide, black circles illustrate truncating mutations, green circles illustrate missense mutations. Domain colors as described in E.

Supplementary Figure 4

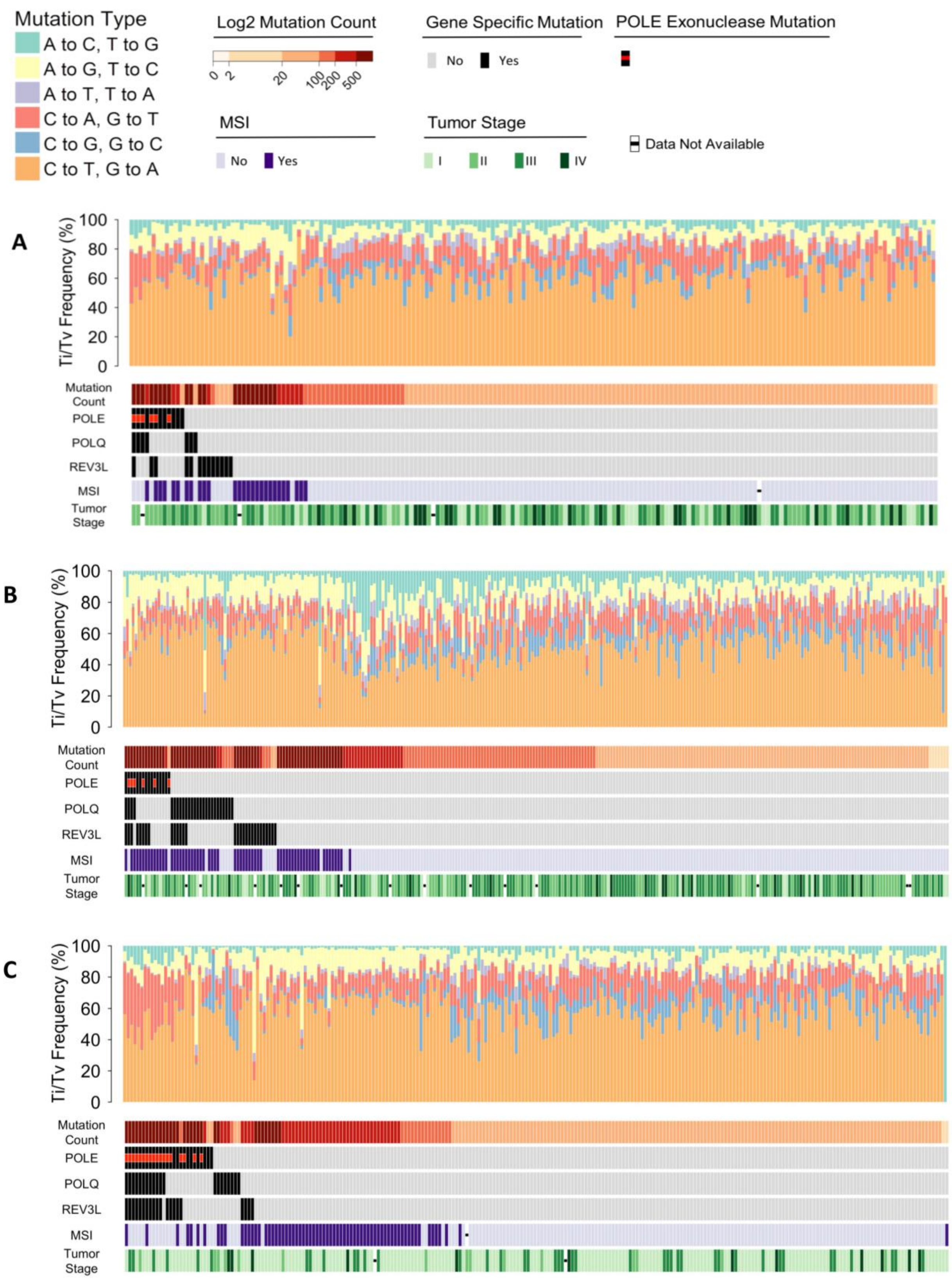

Supplementary Figure 4. ***Relationships between mutation spectrum and mutation counts, POLE, POLQ and REV3L exome mutations, MSI and tumor stage of individual cases.*** Each bar represents an individual tumor for all CORE, STAD, and UCEC in the TCGA data set obtained through cBioportal. The panels are **A)** CORE, **B)** STAD and **C)** UCEC.

Supplementary Figure 5

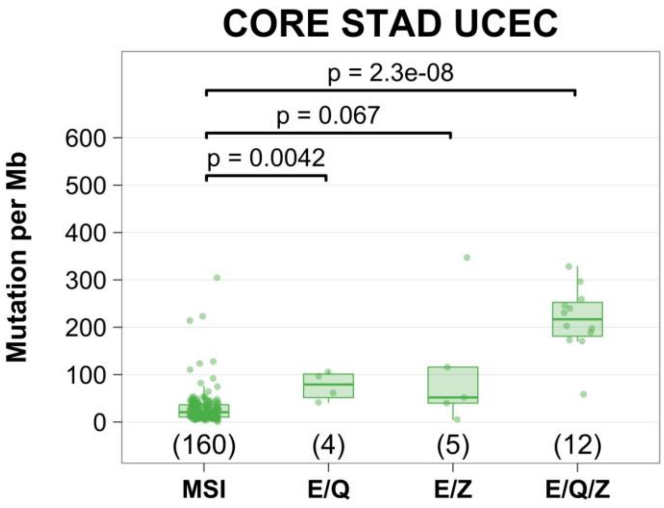

Supplementary Figure 5. Mutation rates per Mb (y-axis) of CORE, STAD and UCEC cases with MSI and E/Q, E/Z and E/Q/Z (x-axis) mutations. Comparisons between groups are significant ($p < 0.05$)

Supplementary Figure 6

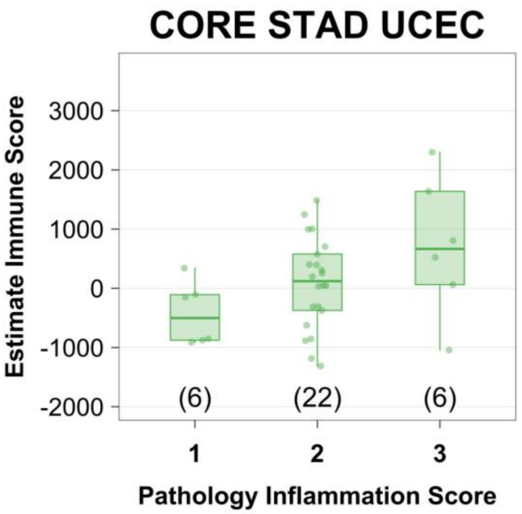

Supplementary Figure 6. *Relationship between pathology inflammation score and ESTIMATE immune scores for CORE, STAD, and UCEC.* Representative tumors of neither POLE nor POLQ mutated, E&Q mutated and MSI were selected. The number of cases is shown in parentheses. Semi-quantitative inflammation scores were evaluated by pathologist using digital images of these samples available in cBioportal. X-axis shows the level of pathology inflammation score. Y axis depicts ESTIMATE immune score.

Supplementary Figure 7

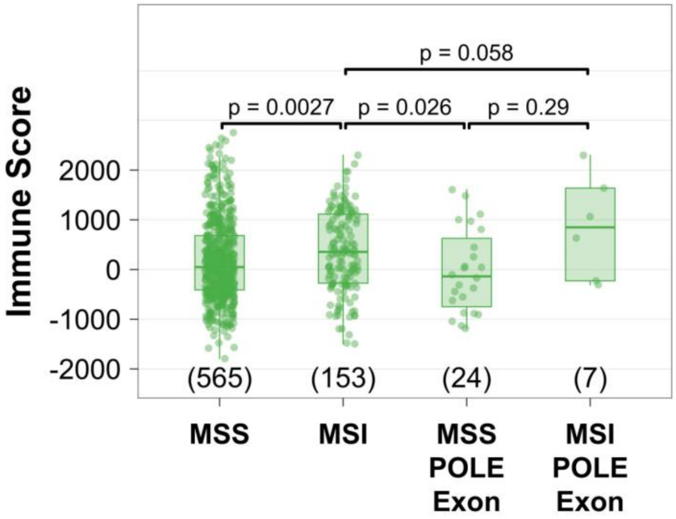

Supplementary Figure 7. *ESTIMATE immune scores in colorectal (CORE), endometrial (UCEC) and stomach (STAD) cancers*. MSS and MSI groups displayed a significant difference in immune scores (p = 0.0027). The MSS group of tumors with mutations in the POLE exonuclease domain displayed significantly lower immune scores than the MSI group (P = 0.028).

Supplementary Table 1

Number of cases with POLE, POLQ and/or REV3L Mutation

	POLE	POLQ	REV3L	E/Q	E/Z	Q/Z	E/Q/Z	Total
**BLCA**	5	6	8	0	0	0	0	127
**BRCA**	8	10	12	0	0	1	1	963
**CESC**	5	9	2	1	0	1	0	191
**CORE**	7	1	8	4	2	2	2	220
**DLBC**	0	4	2	2	0	0	2	48
**HNSC**	6	12	7	0	0	1	0	279
**KIRC**	4	5	1	0	0	0	0	415
**LIHC**	3	7	7	0	0	0	0	193
**LUAD**	10	11	7	1	1	1	0	230
**LUSC**	7	14	1	0	0	2	0	178
**PAAD**	1	1	2	0	0	0	1	145
**SARC**	5	4	2	1	0	0	0	240
**SKCM**	11	18	11	3	3	2	0	278
**STAD**	7	16	15	1	5	6	3	287
**UCEC**	9	8	4	1	5	0	11	242

Supplementary Table 1. Number of cases with POLE, POLQ, Z/REV3L, or multiple exome mutations in the PANCAN cohort. The number of cases with E/Q, E/Z, and E/Q/Z mutations are highest for CORE (n = 8), STAD (n = 9), and UCEC (n = 17).

Supplementary Table 2

A. Frequency of high and low stage in POL mutation groups. P = 0.42

	Others	POLE	E/Q	E/Z	E/Q/Z
**High**	255	8	0	3	5
**Low**	418	15	5	8	11

B. Frequency of MSI in POL mutation groups. P < 0.001

	Others	POLE	E/Q	E/Z	E/Q/Z
**MSI**	133	14	2	7	4
**MSS**	557	9	4	5	12

C. Frequency of MSI in POL mutation groups in 3 cancer types.

	CORE (p = 0.003)					STAD (p < 0.001)					UCEC (p = 0.68)				
	Others	POLE	E/Q	E/Z	E/Q/Z	Others	POLE	E/Q	E/Z	E/Q/Z	Others	POLE	E/Q	E/Z	E/Q/Z
**MSI-H**	22	4	1	1	0	49	6	1	5	2	62	4	0	1	2
**MSS**	182	3	3	1	2	222	1	0	0	1	153	5	1	4	9

D. Frequency of MSI in groups of total mutation levels. P < 0.001

	Total Mutation Level		
	High	Intermediate	Low
**MSI**	144	18	1
**MSS**	46	367	182

Supplementary Table 2. Contingency table showing number of cases of colorectal, endometrial and stomach cancers in each category. The χ^2 p-values indicate the difference in frequencies. **A)** High (3&4) and low (1&2) tumor stage versus POLE, E/Q, E/Z and E/Q/Z mutation group. **B)** MSI and MSS in groups with POLE, E/Q, E/Z and E/Q/Z mutations. **C)** Cancer type subdivision of MSI and MSS status with POLE, E/Q, E/Z and E/Q/Z mutations. **D)** Mutation quartiles versus MSI and MSS status.
